# Proanthocyanidins isolated from the leaves of *Ficus glomerata* evaluated on the activities of rumen enzymes: *in vitro* and *in silico* studies

**DOI:** 10.3389/fchem.2024.1359049

**Published:** 2024-02-06

**Authors:** Suman Lata, Pushpendra Koli, Sultan Singh, Brijesh Kumar Bhadoria, Umesh Chand, Dinesh Kumar Yadav, Thamer Al-Shuwaili, Yonglin Ren

**Affiliations:** ^1^ ICAR-Indian Grassland and Fodder Research Institute, Jhansi, India; ^2^ College of Environmental and Life Sciences, Murdoch University, Murdoch, WA, Australia; ^3^ Department of Microbiology, Central University of Punjab, Bathinda, India; ^4^ ICAR-Indian Institute of Soil Science, Bhopal, India; ^5^ College of Agriculture, Kerbala University, Kerbala, Iraq

**Keywords:** *Ficus glomerata*, proanthocyanidins, phenolic compounds, rumen fermentation, drug score prediction

## Abstract

Two new proanthocyanidins (2S:3S)-(−)-epicatechin-(4α→8)4-(2R:3R)-(+)-catechin (Compound 1) and (2R, 3R)-3-O-galloyl-(+)-catechin (4β→8)3-(2R, 3R)-3-O-galloyl-(+)-catechin (Compound 2) were isolated from *Ficus glomerata* and characterized by ultraviolet spectroscopy (UV), proton nuclear magnetic resonance (^1^H NMR), ^13^C NMR, and heteronuclear multiple bond correlation . The bioactivity and drug scores of isolated compounds were predicted using OSIRIS property explorer applications with drug scores of 0.03 (compound 1) and 0.05 (compound 2). Predictive drug scores provided an indication of the compounds’ potential to demonstrate desired biological effects. Furthermore, the newly discovered proanthocyanidins tended to interact with protein due to their chemical structure and molecular conformation. With the aim of maintaining this focus, compounds 1 and 2 were subjected to *in vitro* testing against ruminal enzymes to further explore their potential impact. Both compounds showed significant inhibition activities (*p < 0.01*) against glutamic oxaloacetic transaminase in both protozoa and bacterial fractions, with an effective concentration (EC_50_) of 12.30–18.20 mg/mL. The compounds also exhibited significant inhibition (*p < 0.01*) of ruminal glutamic pyruvic transaminase activity, with EC_50_ values ranging from 9.77 to 17.38 mg/mL. Furthermore, the inhibition was recorded in R-cellulase between EC_50_ values of 15.85 and 23.99 mg/mL by both compounds. Additionally, both compounds led to a decrease in protease activity with increasing incubation time and concentration. In conclusion, the results indicate that these novel proanthocyanidins hold the potential to significantly impact rumen enzyme biology. Furthermore, their promising effects suggest that they could be further explored for drug development and other important applications.

## 1 Introduction

The field of plant secondary metabolites, specifically polyphenols, has emerged as a crucial area of research due to its profound impact on animal health. Polyphenol compounds, readily available in various sources such as vegetables, fruits, cereals, herbs, forage legumes, and spices, belong to major classes including flavonoids, non-flavonoids, and tannins (Bento et al., 2017; Durazzo et al., 2019). Proanthocyanidins (PAs) or condensed tannins are widespread in the plant kingdom and are known to possess a greater affinity for dietary and salivary protein, digestive enzymes, and carbohydrates (Mueller-Harvey and McAllan, 1992; Zhang et al., 2023). PAs play a vital role in ruminants’ nutrition due to their ability to interact with dietary protein. Additionally, to some extent, PAs can also have an affinity to interact with metals, amino acids, carbohydrates, digestive enzymes, and microbes (Jonker and Yu, 2017). The presence of these molecules in the tree leaves could be linked with the anti-quality or anti-nutritional properties in feeds (Deshpande et al., 1984; Mitaru et al., 1984; Katoch, 2022) when the levels of PA are high (Barry and Duncan, 1984; Wiseman and Cole, 1988); however, their low-level presence in feeds is able to prevent bloating in cattle (Waghorn and Jones, 1989; Lees, 1992). It is now recognized that a low concentration of proanthocyanidins in ruminant diets can also be highly beneficial in many other ways, such as reducing the effects of parasites in the gastrointestinal tract (Czochanska et al., 1979; Besharati et al., 2022), reducing problems relating to fly strike (Koupai-Abyazani et al., 1992), and, paradoxically, improving protein availability (Hagerman and Carlson, 1998; Hagerman et al., 1998). However, having different chemical structures, not all proanthocyanidins exhibit the same kind of beneficial effects on ruminants’ nutrition. Because of this, there is a difference in nature varying with changes of genus, species, and varieties. Stereochemistry is also one important characteristic for bringing variations in the function of proanthocyanidins. Therefore, among the polyphenolic compounds, proanthocyanidins have received significant attention for their role in ruminants’ nutrition. Thus, the function of polyphenolic is not only dependent on the concentration but also on the chemical structure and composition. Due to this, the structure-activity relationship of polyphenols is imperative in defining the nutritional impact. In this backdrop, investigations were undertaken on polyphenolics of leaves of the most common tree *Ficus glomerata* Roxb (Fam. Moraceae) locally called “Gular” and “Udumbare” (Sanskrit), and it is also known as “Cluster fig”. *Ficus glomerata* Roxb (Fam. Moraceae) is one of the most popular species of genus *Ficus*, abundantly found throughout India, especially in Rajasthan, Bundelkhand, Uttar Pradesh, and Southeast Punjab. This study revealed a research gap in the limited exploration of underlying molecular mechanisms for the positive effects of polyphenols on animal health and rumen enzymes, highlighting the need for comparative studies with compounds from different plant sources to contextualize and enhance the significance of the findings. In a noteworthy contribution to this field, our work focused on characterizing two novel compounds derived from the leaves of *F. glomerata*. Through rigorous evaluation using *in silico* experiment, we assessed their drug score and investigated their inhibitory effects on rumen enzyme activity.

## 2 Materials and methods

### 2.1 Chemicals and reagents

The chemical reagents and solvents including tannic acid, gallic acid, 2S, 3S (−)-epicatechin, 2R, 3R (+)-gallocatechin, 2S, 3S (−)-epicatechin-3-O-gallate, acetone, methanol, butanol, acetic acid, and Sephadex LH-20 of analytical grade were procured from Sigma, United States.

### 2.2 Isolation of proanthocyanidins

The leaves sample (Approximately 5 kg) of *F. glomerata* was collected from the Central Research Farm of ICAR-Indian Grassland and Fodder Research Institute, Jhansi, India (25.5113° N, 78.5337° E), in monsoon season. The leaves were dried at 60°C to the stage where there was no further reduction in the weight and then dried leaves were grounded to a powder and passed through a 1 mm sieve. The fat content was removed by soaking overnight in hexane (Ibrahim et al., 2013) and 1.5 kg of defatted leaves powder was subjected to extraction by 70% aqueous acetone containing 0.2% ascorbic acid in the Soxhlet extractor. After Soxhlet extraction, the solvent was removed under reduced pressure in a rotatory evaporator at 40°C and suspended in 2 L distilled water for 12 h. The remaining aqueous phase was washed with diethyl ether and ethyl acetate and then the leftover extract (320 g) was chromatographed on the Sephadex LH-20 column. The column was eluted sequentially with 50% aqueous methanol followed by 70% aqueous acetone and then with pure distilled water. The fractions eluted with 50% aqueous methanol were combined owing to their similarity in thin layer chromatography (TLC) and purified by preparative thin layer chromatography (TLC-prep.) and preparative layer chromatography (PLC) over cellulose plates using solvent system pure distilled water to yield light brown solid amorphous compounds 1 and 2 m, which showed a pinkish color. The pictures of the dried powder of both compounds are given in the (Supplementary Figure S1).

### 2.3 Characterization and structure determination

Melting points (m. p.) of compounds 1 and 2 were determined using a Bockmonoscope. A UNCAM UV/vis spectrophotometer (Newington, United States) was used to record UV spectra. Mass spectra were determined on a Jeol mass spectrophotometer (Tokyo, Japan). ^1^H and ^13^C NMR spectra were obtained on Bruker DRX-300 spectrophotometer (Fallanden, Switzerland) with tetramethyl silane as an internal standard, and the heteronuclear multiple bond correlation (HMBC) was measured using a standard pulse sequence. High-performance liquid chromatography (HPLC) was performed using a Shimadzu model LC-8A. To get the circular dichroism (CD) spectrum, the facility at the Department of Pharmacognosy, University of Mississippi, United States, was used. The spectrums for both compounds are presented in the (Supplementary Figure S2). TLC, column chromatography, and paper chromatography (PC) were performed on precoated Si GF^256^, Si gel (60–120 Mesh, Merck India), Sephadex LH-20 (Sigma, United States ), and Whatman paper to characterize both compounds 1 and 2.

### 2.4 Qualitative phytochemical investigation

Compounds 1 and 2 underwent complete acid hydrolysis to study anthocyanidin subunits through Shinoda, vanillin/HCL, and FeCl_3_ tests along with TLC and PC profiling (Jain et al., 1989). To determine monomeric units, compounds 1 and 2 were independently treated with phloroglucinol in the presence of 100 mL of 1% HCl in 50% aqueous methanol in a 250 mL Round Bottom flask for 48 h. After drying the solvent, the product was diluted with H_2_O and extracted with ethyl acetate followed by evaporation. The dried product was dissolved in 80% methanol and subjected to quantitative analysis by 2D HPTLC (TLC plate cellulose 20 × 20 cm; solvent of tertiary butanol: acetic acid: water at 3:1:1) and HPLC equipped with UV/v detector at 280 nm and RP ODS column (25 cm × 4 mm, id) at an ambient temperature with solvents of acetic acid (1%) (A) and methanol (B) at 1 mL/min.

### 2.5 In silico studies

*In silico* studies were performed using open-source software for the virtual screening of the two novel compounds. Drug score value qualifies the overall potential of a compound as a drug candidate. OSIRIS property explorer (a web-based software) was used to predict drug score by considering toxicity risks, partition coefficient between n-octanol and water (cLogP), solubility (logS), molecular weight (Mw), total polar surface area (TPSA), number of hydrogen acceptor and donor, number of rotatable bonds, and toxicity risks (OSIRIS, 2023).

### 2.6 *In vitro* ruminal enzyme activity

An adult sheep was selected for sampling from the small ruminant unit of the Plant Animal Relationship Division of IGFRI, Jhansi. Rumen liquor was collected before feeding. It was obtained through the mouth using a perforated plastic tube with light suction in a 0.5 L capacity pre-warmed thermos (Singh and Kundu, 2010). A ruminal cellulase extract was prepared from the collected rumen liquor and the effect of isolated compounds on its activities was estimated according to a described method (Mandels and Weber, 1969). A protocol for determining the activity of the intra-cellular enzymes’ glutamic pyruvic transaminase (GPT) and glutamic oxaloacetic transaminase (GOT) was used (Yatzidis, 1960), while the bacterial and protozoal fractions of the rumen liquor were obtained, and then, the separation of bacteria and protozoal-rich enzyme extracts in a 0.1 M phosphate buffer of pH 6.8 was carried out according to our published methods (Singh and Kundu, 2010; Koli et al., 2022; Singh et al., 2022). To measure proteolytic enzyme activities, the concentration of protein in enzyme extracts was estimated according to a previous method (Lowry et al., 1951). The proteolytic activity of isolated compounds was determined by estimating undigested protein from casein (Blackburn and Hobson, 1960; Kumar and Singh, 1984).

### 2.7 Statistical analysis

For the statistical analysis, Microsoft Excel 2016 and R (R-4.2.3) software were used. To evaluate enzymatic activities, Analysis of Variance (ANOVA) was performed using R, and significant differences in means were determined at *p < 0.01* using *post hoc* analysis with Tukey’s test.

## 3 Results and discussion

### 3.1 Characterization of isolated compounds 1 and 2

**FIGURE 1:**
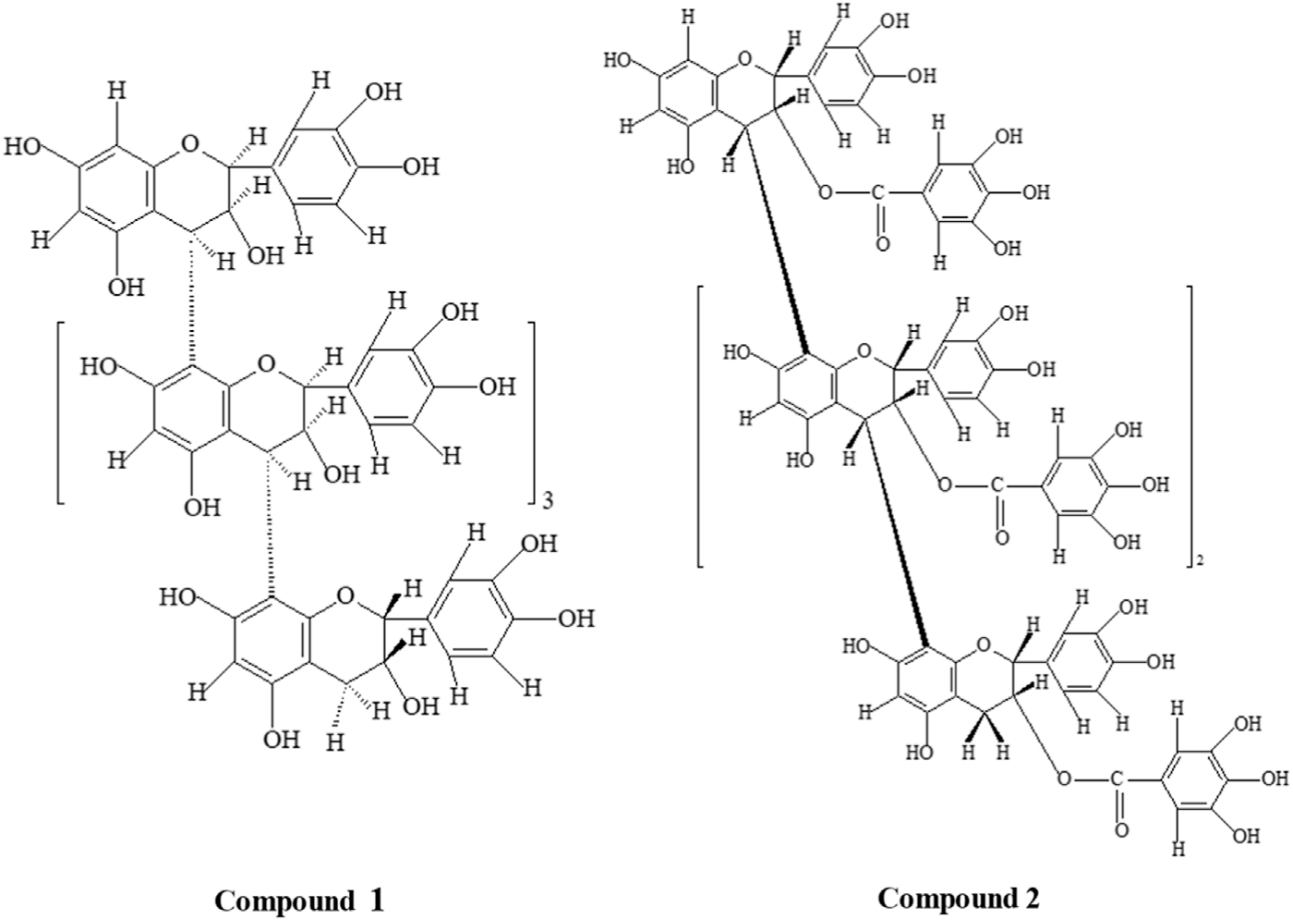
Structures of compounds 1 [(2*S*:3*S*)-(−)-epicatechin-(4α→8)_4_-(2*R*:3*R*)-(+)-catechin] and 2 [(2*R*, 3*R*)-3-O-galloyl-(+)-catechin-(4β→8)_3_-(2*R*, 3*R*)- 3-O-galloyl-(+)-catechin].

Compound 1: Light brown solid, m.p. 260°C–62°C, and UV(MeOH) λ_max_ 282nm; FAB-MS [M + H]^+^ 1,443, C_75_H_62_O_30_; m/z 1,289, 1,153, 865, 577, 289, and 151; CD spectral data, CD at 225.8 nm CD [mdeg] = −10.182, at 251.9 nm CD [mdeg] = 8.37356, at 270.4 nm CD [mdeg] = −8.1183, and 306.5 CD [mdeg] = 3.30982. ^1^H NMR, ^13^C NMR, and HMBC data are given in Table 1, and the structure is depicted in Figure 1.

**TABLE 1:** ^1^H and ^13^C NMR and HMBC spectroscopic data for compound 1 in DMSO-d6 (δ, ppm, J/Hz).

Position	δ_H_	δ_C_	HMBC (J)
C-2 u, m	5.175 (4H, d, *J* = 4.2 Hz)	76.7	1′^2^
C-2 t	4.141 (1H, d, *J* = 9.3 Hz)	81.8	1′^2^
C-3 u, m	3.418 (4H, dd, *J* = 4.5, 4.2 Hz)	73.7, 72.1	
C-3 t	3.822 (1H, m)	65.1	
C-4 u, m	4.974 (4H, d, *J* = 6.9 Hz)	38.3	
C-4 t	3.984 (1H, dd, *J* = 9.6, 9.9 Hz)	30.2	3′^2^
C-5		156	
C-6 u, m	6.174 (1H, d, *J* = 3.3 Hz) 6.009(4H, s, H-6 m, t)	96.3	
C-6 t		96.3	
C-7		160.8	
C-8 u	6.301 (1H, d, *J* = 3.3 Hz)	99.9	6 ^3^
C-8 m, t		102.5, 103.3	
C-9		109.6	
C-10		151.2	
C-1′		134.0	
C-2′	6.743(5H, s)	120.6	
C-3′		145.0	
C-4′		148.7	
C-5′	6.957(5H, d, *J* = 7.8 Hz)	117.8	
C-6′	6.834 (5H, d, *J* = 7.8 Hz)	118.0	

u-upper unit; m-middle unit; t-terminal unit, HMBC- 2D, heteronuclear multiple bond correlation.

Compound 2: Pinkish microcrystalline substance m. p. 266°-8°; UV(MeOH) λ_max_ 284nm; FAB-MS [M + H]^+^1763, C_88_H_66_O_40_; m/z 1,457, 1,321, 881, 441, and 303; ^1^H NMR, ^13^C NMR, and HMBC data are given in Table 2, and the structure is depicted in Figure 1.

**TABLE 2:** ^1^H and ^13^C NMR and HMBC spectroscopic data for compound 2 in DMSO-d6 (δ, ppm, J/Hz).

Position	δ_H_	δ_C_	HMBC (J)
C-2 u, m	4.479 (3H, d, *J* = 7.2 Hz)	81.2, 81.1	1′^2^
C-2 t	4.479 (1H, d, *J* = 7.2 Hz)	80.7	1′^2^
C-3 u, m	3.925 (3H, dd, *J* = 6.9, 6.9 Hz)	73.0, 70.0	
C-3 t	3.340 (1H, m)	65.9	2^2^
C-4 u, m	4.628 (3H, d, *J* = 4.5 Hz)	39.7, 39.5	
C-4 t	3.583 (1H, dd, *J* = 5.4, 4.8 Hz)	29.1	
C-5		156.2	
C-6 u, m	5.878 (1H, d, *J* = 1.8Hz, u)	94.0,95.2	8 ^3^
	6.186 (s, m)		
C-6 t	6.186 (s)	95.2	8 ^3^
C-7		156.5	
C-8 u	5.679 (1H, d, *J* = 1.8 Hz)	99.2	6 ^3^
C-8 m, t		109.3, 109.2	
C-9		155.4, 155.2	
C-10		114.5	
C-1′		130.3, 130.7	
C-2′	6.921 (4H, d, *J* = 2.7 Hz)	118.3	1′,3′^2^
C-3′		145.8	
C-4′		145.0	
C-5′	6.660 (4H, d, *J* = 6.6 Hz)	118.5	3′^3^ *J* and 4′^2^
C-6′	6.716 (4H, dd, *J* = 6.6, 3.3 Hz)	115.3	3′^3^
C-1″		139.2 u, m	
		139.4 t	
C-2″	6.179 (4H, d, *J* = 1.2 Hz)	115.2	
C-3″		145.8	
C-4″		144.9	
C-5″		149.9	
C-6″	6.111 (4H, d, *J* = 1.5 Hz)	1,114.6	
C-7″		164.6	

u-upper unit; m-middle unit; t-terminal unit, HMBC- 2D, heteronuclear multiple bond correlation.

Both isolated compounds (1 and 2) responded to the characteristic reactions of proanthocyanidin (Ishimaru et al., 1987; Geiss et al., 1995). The appearance of strong UV_(MeOH)_ absorption at 282 and 284 nm supported and confirmed the proanthocyanidin nature of compounds 1 and 2, respectively (Kennedy and Jones, 2001). Complete acid hydrolysis of compound 1 with n-BuOH-HCl (95:5) furnished cyanidin (R_f_ 52), as only anthocyanidin pigment characterized by co-paper chromatography using solvent system forestal indicated compound 1 to be oligomeric procyanidin composed of catechin/epicatechin units, whereas compound 2 furnished cyanidin and gallic acid (R_f_ 40), which showed compound 2 as oligomeric procyanidin, composed of catechin/epicatechin units with galloyl moieties. The physico-chemical properties of isolated compounds along with the standard are illustrated in Table 3. The protonated fast atom bombardment-mass spectrometry (FAB-MS) of compound 1 afforded a molecular ion peak (M + H) at 1,443, consisting of C_75_H_62_O_30_ furnished significant molecular ion fragments at m/z 1,289, 1,153, 865, 577, 289 and 151 due to *retro-Diels-Alder* (RDA) cleavage, which confirmed the presence of catechin/epicatechin in upper, middle and terminal position of molecule (Self et al., 1986). Furthermore, the appearance of molecular fragments at 151 and 1289 verified structure 1 as homogeneous oligomeric catechin and epicatechin. In the protonated FAB-MS spectrum of compound 2, the M + H peak is observed at m/z 1763, corresponding to the molecular formula C_88_H_66_O_40_. This peak indicates the presence of molecular species resulting from Reactive Desorption and Attachment (RDA) at m/z values of 1457, 1321, 881, 441, and 303. Notably, the confirmation of the galloyl moiety is evident in both the upper and terminal units, as reflected by the peaks at m/z 303 and 1457. Furthermore, the peaks appeared at m/z; 1,321, 881, and 441 were resultant of carbon-carbon inter flavonoids bond cleavage which confirmed 2 as homogeneous oligomeric 3-O-galloyl-(+)-catechin.

**TABLE 3:** Physico–chemical properties of isolated compounds.

Compounds	Iupac name	Solubility	Melting point (°C)	Molecular formula
Compound 1	(2*S*:3*S*)-(−)-epicatechin-(4α→8)_4_-(2*R*:3*R*)-(+)-catechin	Water	260–62	C_75_H_62_O_30_
Compound 2	(2*R*, 3*R*)-3-O-galloyl-(+)-catechin-(4β→8)_3_-(2*R*, 3*R*)- 3-O-galloyl-(+)-catechin	Water	266–68	C_88_H_66_O_40_
Tannic acid	[2,3-dihydroxy-5-[[(2R,3R,4S,5R,6S)-3,4,5,6-tetrakis [[3,4-dihydroxy-5-(3,4,5-trihydroxybenzoyl) oxybenzoyl]oxy]oxan-2-yl]methoxycarbonyl]phenyl] 3,4,5-trihydroxybenzoate	Alcohol, acetone, Water	200	C_76_H_52_O_46_
Gallic acid	3,4,5-trihydroxybenzoic acid	Water	258–265	C_7_H_6_O_5_
(−)-Epicatechin	(2*S*,3*S*)-2-(3,4-dihydroxyphenyl)-3,4-dihydro-2H-chromene-3,5,7-triol	Water, Alcohol	235–237	C_15_H_14_O_6_
(+)-Gallocatechin	(2*R*,3*S*)-2-(3,4,5-trihydroxyphenyl)-3,4-dihydro-2*H*-chromene-3,5,7-triol	Water	189–191	C_15_H_14_O_7_

Both compounds further underwent ^13^NMR characterization. The chemical shifts observed in spectrums of regions A (30–90 ppm) and B (90–160 ppm) indicated the structural composition of oligomeric flavan-3-ol (Czochanska et al., 1979). For compound 1 in region A, out of 16 aliphatic carbons, five oxygenated methine carbons appeared for C-3 of u, m, and t at δ73.7, δ72.1, and δ65.1. The up-field resonance at δ38.3 and δ30.2 ppm were attributed to the C-4 of the upper, extender, and terminal flavonoid units with 3,4 *cis* configuration. The upper and extender units of the molecule were predominant in 2,3 *cis* configuration as indicated by the up-field resonance of the heterocyclic ring carbon at δ76.7, whereas the terminal unit of the molecule possessed 2,3 *trans* configuration supported by the absence of the corresponding downfield signal for the terminal unit (δ81.8), and 2,3 *cis* and 3,4 *cis* stereochemistry of upper and extender units was further confirmed by downfield signal which appeared at δ76.7 in ^13^C NMR for C-2 (u,m) (Foo et al., 1997). Furthermore, the observance of chemical shift at δ81.8 for C-2 (t) supported the 2,3 *trans* 3,4 *cis* configuration in the terminal unit. The absence of the γ-gauche effect in ^13^C NMR for C-4 in ring-C relative to C-2 was strongly indicative of 2,4-*cis* orientation with 4*S* configuration in procyanidin molecules (Porter et al., 1982). The circular dichroism spectrum of compound 1 showed a negative cotton effect at 225.8 nm (CD [medg] = −10.182), which finally led to a decision of the 4*S* configuration of the proton with α linkage (Nunes et al., 1989). Region B of the spectrum displayed characteristic chemical shifts for 21 aromatic methine carbons at δ96.3 (C-6 u, m, t), δ99.9 (C-8 u), δ102.5 (C-8 m), δ103.3 (C-8 t), δ120.6 (C-2′ u, m, t), δ117.8 (C-5′ u, m, t), and δ118.0 (C-6, u, m, t) and hydroxyl-substituted carbons at δ145.0 and δ148.7 were indicative for C-3′ and C-4′, respectively, of the u, m, and t units. The quaternary carbons were displayed by δ134.0 (C-1′), δ109.6 (C-9), and δ151.2 (C-10). The oxygenated carbons of A ring at C-5 and C-7 displayed at δ156.8 and δ160.8 in the u, m, and t units, respectively. Whereas compound 2 exhibited 13 aliphatic carbons in region A, in which four oxygenated methine carbons appeared at δ73.0, δ70.0, and δ65.9 for C-3 of upper, extender, and terminal units (Harborne and Mabry, 2013). The up-field signal at δ39.7 and δ39.5 was attributed to the tertiary (methine) C-4 carbons of upper and extender units with 3,4 *trans* configuration indicated in distortionless enhancement by polarization transfer (DEPT) 90° and 135° experiment, whereas the secondary C-4 carbon of terminal unit appeared at δ29.1 in ^13^C NMR spectrum and in DEPT 90° and 135° experiment (Kemp, 2017). Furthermore, the appearance of up-field resonance in ^13^C NMR spectrum and DEPT 90° and 135° experiment at δ81.2, δ81.1, and δ80.7 for C-2 of u, m, and t indicated 2,3 *trans* configuration in upper, extender, and terminal units, respectively (Foo et al., 1997). The observance of the γ-gauche effect in ^13^C NMR for C-4 in ring-C at δ39.7 and δ39.5 relative to δ81.2 and δ81.1 for C-2 in upper and extension units further corroborated 2,4-*trans* orientation in this procyanidin molecule (Cai et al., 1991). Region B of the spectrum exhibited characteristic chemical shifts for 17 aromatic methine carbons at δ94.0 (C-6 u), δ95.2 (C-6 m, t), δ99.2 (C-8 u), δ109.3 (C-8 m), δ109.2(C-8 t), δ115.3 (C-6′ u, m, t), δ118.3 (C-2′ u, m, t), and δ118.5 (C-5′ u, m, t) duly supported by DEPT experiments. Hydroxy-substituted carbons C-3′ and C-4′ of the upper, extender, and terminal units were displayed at δ145.8 and δ145.0 along with four quaternary carbons at δ130.3 and δ130.7 of C-1’ of the upper, extender, and terminal units (Porter, 2017). The remaining A ring oxygenated-aromatic carbons appeared at δ156.2 and δ156.5 for C-5 and C-7 of the upper, extender, and terminal units (Porter et al., 1982). The chemical shifts at δ155.4, δ155.2, and δ114.5 were due to C-9 and C-10 of u, m, and t respectively. The additional carbon signals at δ139.2 (C-1″ u, m), δ139.4 (C-1″ t), δ115.2 (C-2″ u, m, t), δ145.8 (C-3″ u, m, t), δ144.9 (C-4″ u, m, t), δ149.9 (C-5″ u, m, t), and δ114.6 (C-6″ u, m, t), respectively, confirmed the presence of galloyl moiety in the molecule (Nonaka et al., 1983). The methine carbons of galloyl moiety C-2″ and C-6″ were also supported by DEPT 90° and 135° experiment. The quaternary carbon of galloyl moiety was displayed at δ164.6 for C-7″.

In ^1^H NMR of compound 1 (DMSO-d_6_), the appearance of two doublets at δ6.301 (1H, d, *J* = 3.3 Hz) and δ6.174 (1H, d, *J* = 3.3 Hz) indicated *meta* coupled C-8 and C-6 protons of upper flavonoids unit with C-4→C-8 linkage with extension unit (Geiss et al., 1995) as affirmed by ^3^ *J* coupling of H-8 to C-6 in HMBC. The presence of two doublets at δ5.175 (4H, d, *J* = 4.2 Hz) and δ4.974 (4H, d, *J* = 6.9 Hz) and a double doublet at δ3.418 (4H, dd, *J* = 4.5, 4.2 Hz) formed the AMX system for C-2, C-4, and C-3 position of the upper and extension units. The doublet at δ4.974 with a coupling constant of 6.9 Hz corroborated α-linkage at C-4 (Cai et al., 1991). The low coupling constant (*J* = 4.2 and 4.5 Hz) for C-2 and C-3 was indicative of 2,3 *cis* orientations. The chemical shifts at δ4.141 as a doublet (1H, d, *J* = 9.3 Hz), multiplet at δ3.822 (1H, m), and doublet at δ3.984 (1H, dd, *J* = 9.6, 9.9 Hz) formed the AMX_2_ system for C-2, C-3, and C-4 of the terminal unit, which was further confirmed by the observance of ^2^ *J* coupling of H-4 (t) to C-5 and H-2 (u, t) to C-1′ in HMBC. The large coupling constant (*J* = 9.3 Hz) for C-2 and C-3 allowed us to infer 2,3 *trans* configuration in the terminal unit (Foo et al., 1997). The singlet at δ6.009 (4H) was indicative of the C-6 proton of the extender and terminal units. The signal arising as a singlet at δ6.743 (5H) was indicative of the presence of a free proton at C-2′ of the B ring of the upper, extension, and terminal units, respectively. The *ortho-*coupled C-5′ and C-6′ protons of the B ring of the upper, extension, and terminal units were visible in the form of doublets at δ6.957 (5H, d, *J* = 7.8 Hz) and δ6.834 (5H, d, *J* = 7.8 Hz), respectively. The availability of protons at C-2′, C-5′, and C-6′ in the B ring suggested the presence of (−)-epicatechin unit in upper and extender units of molecule with 2,3 *cis* configuration as confirmed by the low coupling constant (*J* = 4.2 Hz) and (+)-catechin unit in terminal unit with 2,3 *trans* configuration as supported by the observed large coupling constant (*J* = 9.3 Hz). Whereas the ^1^H NMR (DMSO-d_6_) of compound 2 displayed two doublets at δ4.479 (3H, *J* = 7.2 Hz) and δ4.628 (3H, *J* = 4.5 Hz) and a double doublet at δ3.925 (3H, *J* = 6.9, 6.9 Hz), forming the AMX system (Silverstein and Bassler, 1962) for C-2, C-4, and C-3 positions of the upper and extension units. Furthermore, the ^1^H NMR appearance of a doublet at δ4.628 with a coupling constant of 4.5 Hz corroborated β-linkage at C-4 (Cai et al., 1991). The 2,3-trans orientation in the molecule was evident from the large coupling constant (*J* = 7.2, 6.9 Hz) for C-2 and C-3 (Foo and Porter, 1978; Ishimaru et al., 1987; Foo et al., 1997). Resonance at δ4.479 as doublet (1H, *J* = 7.2 Hz), multiplet at δ3.340 (1H), and double doublet at δ3.583 (1H, *J* = 5.4, 4.8 Hz) formed the AMX_2_ system of C-2, C-3, and C-4 of terminal unit, which was further corroborated by 2,3 *trans* configuration in the terminal unit of molecule (Foo and Porter, 1978; Ishimaru et al., 1987; Foo et al., 1997). These positions were further affirmed by the observance of ^2^ *J* coupling of H-3 (t) to C-2 (t) and H-2 (u, m, t) to C-1′ (u, m, t) in the HMBC spectrum. The appearance of two doublets at δ5.878 (1H, *J* = 1.8 Hz) and δ5.679 (1H, *J* = 1.8 Hz) were suggestive of *meta*-coupled C-6 and C-8 protons of upper flavonoid unit with C-4 to C-8 linkage with the extension unit (Geiss et al., 1995). This further ascertained their positions by significant ^3^ *J* coupling of H-6 to C-8 and H-8 to C-6 in the HMBC spectrum. The chemical shifts as a singlet at δ6.186 (3H) was an indication for the C-6 proton of extension and the terminal unit, inferred by the observance of ^3^ *J* coupling with C-8 (m, t). The protons located at C-2′, C-5′, and C-6′ positions of the B ring in the upper, extension, and terminal units were detected as a doublet with chemical shifts at δ6.921 (4H, *J* = 2.7 Hz) and δ6.660 (4H, *J* = 6.6 Hz). Additionally, a double doublet was observed at δ6.716 (4H, J = 6.6, 3.3 Hz). These findings indicate the connectivity pattern, specifically the ^2^ *J* coupling of H-2′ to C-1′ and C-3′, H-5′ to C-4′, and ^3^ *J* coupling of H-5′ to C-3′ and H-6′ to C-4′. This information facilitated the assignment of their respective positions. The availability of free protons at C-2′, C-5′, and C-6′ in B ring suggested the presence of catechin unit in the upper, extension, and terminal units of the molecule (Geiss et al., 1995). The appearance of two *meta*-coupled doublets at δ6.179 (4H, *J* = 1.2 Hz) and δ6.111 (4H, *J* = 1.5 Hz) for C-2″ and C-6″ showed the presence of galloyl moieties in the molecule (Nonaka et al., 1983). Finally, their significant connectivity as ^3^ *J* coupling with C-7″ confirmed their positions.

### 3.2 Structural analysis of isolated compounds through phloroglucinol degradation

The treatment of compounds 1 and 2 with phloroglucinol and 1% HCl in methanol, compound 1**-**yielded (+)-catechin, and (−)-epicatechin-4α-phloroglucinol was examined by 2D high-performance thin-layer chromatography (HPTLC) and high-performance liquid chromatography HPLC. HPLC separation employed a mobile phase comprising a 1% aqueous acetic acid (A) and methanol (B) solvent system. The elution was conducted at a flow rate of 1 mL/min, with a gradual increase of B in A. The percentage composition changed linearly, starting from 0% to 15% in the first 30 min, progressing to 15%–60% between 30 and 45 min, and maintaining at 60% from 45 to 60 min. The HPLC examination of compound 1 showed only two peaks for (+)-catechin (Rt = 40.06 min) with 2*R*:3*R* configuration and (−)-epicatechin-4-phloroglucinol (Rt = 24.38 min) with 2*S*:3*S* configuration, indicating the presence of 2*S*:3*S*-(−)-epicatechin and 2*R*:3*R*-(+)-catechin in extension and terminal units, respectively (Koupai-Abyazani et al., 1992). Compound 1 had undergone solvolysis and resulted in (+)-catechin and (−)-epicatechin. The yield of (−)-epicatechin-4α-phloroglucinol by the cleavage of the chain extension unit suggested C-4→C-8 interflavan linkage in procyanidin B type. Whereas, compound 2 revealed the presence of 3-O-galloyl-(+)-catechin and 3-O-galloyl-(+)-catechin-4β-phloroglucinol with phloroglucinol and 1% HCl in methanol. The cleavage of the chain extension unit with the yield of 3-O-galloyl-(+)-catechin-4β-phloroglucinol suggested C-4→C-8 interflavan linkage in procyanidin B type. These pieces of evidence were adequate to characterize compound 1 as (2*S*:3*S*)-(−)-epicatechin-(4α→8)_4_-(2*R*:3*R*)-(+)-catechin and compound 2 as (2*R*, 3*R*)-3-O-galloyl-(+)-catechin (4β→8)_3_-(2*R*, 3*R*)- 3-O-galloyl-(+)-catechin.

### 3.3 Drug scores prediction by *in silico* studies

The drug score for compounds 1 and 2 were obtained as 0.03 and 0.05, respectively (Table 4). The obtained scores were varied in the range of low risk of undesired behavior, viz., mutagenicity or poor intestinal absorption, and thus indicated drug-conform behavior with a good signal for druglikeness and bioactivity potential (Lata et al., 2023). The drug score combines druglikeness, cLogP (logarithm of partition coefficient), logS (logarithm of solubility), molecular weight, and toxicity risks in one handy value that may be used to judge the compound’s overall potential to qualify for a drug (Verma, 2012). cLogP (octanol/water partition coefficient) could predict the permeability of molecules across the cell membrane, and total polar surface area (TPSA) relates to the hydrogen bonding potential of the molecule and is a predictor of drug transport properties, such as bioavailability, intestinal absorption, and blood–brain barrier penetration. The number of rotatable bonds exhibits the flexibility of the molecule that describes drug absorption and bioavailability properties (Agwom et al., 2019). Additionally, drug scores of compounds 1 and 2 were also compared with tannic acid (0.31), gallic acid (0.27), 2S, 3S (−)-epicatechin (0.89), and 2R, 3R (+)-Gallocatechin (0.35). Furthermore, a low risk of tumorigenic, irritant, and reproductive effects were also depicted.

**TABLE 4:** ORISIS drug scores.

Compounds	cLogP	TPSA	Druglikeness	H Bond acceptor	H Bond donor	Nb stereocenters	Nb rotatable bonds	Drug-score	Solubility
Compound 1	22.24	166.14	−7.55	18	15	5	5	0.03	−18.83
Compound 2	42.74	276.90	−9.38	30	21	3	3	0.05	−34.63
Tannic acid	5.53	777.98	1.60	46	25	5	31	0.31	−7.60
Gallic acid	0.11	97.99	0.12	5	4	0	1	0.27	−0.74
(−)-epicatechin	1.51	110.38	1.92	6	5	2	1	0.89	−1.76
(+)-gallocatechin	1.96	240.99	2.39	13	11	5	3	0.35	−2.76

### 3.4 Determination of effects on ruminal enzymes *in vitro*

**FIGURE 2:**
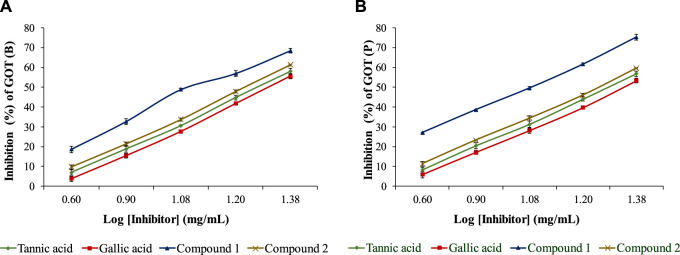
Inhibition effects of isolated compounds along with tannic acid and gallic acid on ruminal glutamic oxaloacetic transaminase, **(A)** bacterial fraction, and **(B)** protozoa fraction.

**FIGURE 3:**
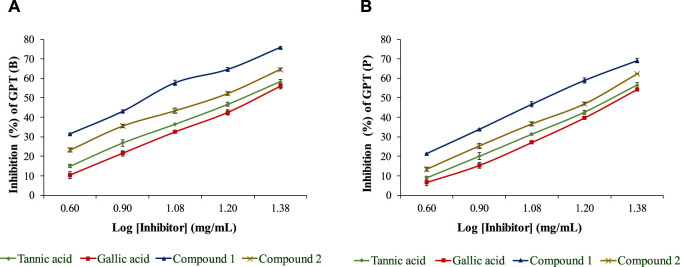
Inhibition effects of isolated compounds along with tannic acid and gallic acid on ruminal glutamic pyruvic transaminase, **(A)** bacterial fraction, and **(B)** protozoa fraction.

**FIGURE 4:**
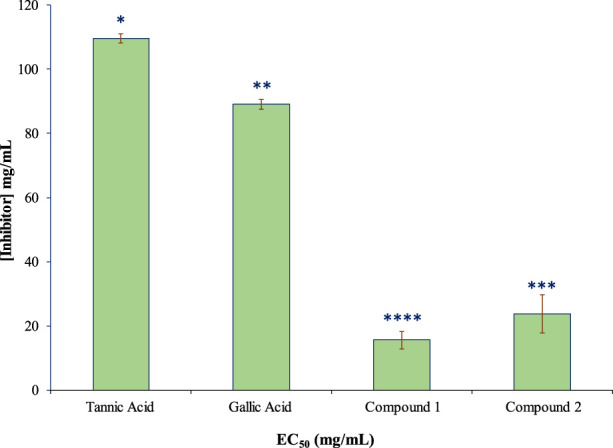
Inhibition effects (EC_50_) of isolated compounds in comparison with tannic acid and gallic acid on ruminal cellulase.

The isolated compounds exhibited inhibitory effects on the ruminal glutamic oxaloacetic transaminase (R-GOT; Figure 2) and glutamic pyruvic transaminase (R-GPT; Figure 3). Compounds 1 and 2 potentially (*p < 0.01*) inhibited R-GOT (protozoal) with an EC_50_ of 13.31 and 18.20 mg/mL, respectively. Whereas, in bacteria fraction, the EC_50_ for compounds 1 and 2 was recorded as 12.59 and 17.38 mg/mL, respectively. In the case of GPT, for the protozoal fraction, compounds 1 and 2 inhibited at an EC_50_ of 11.75 and 17.38 mg/mL, respectively, and in bacterial fraction, EC_50_ was recorded as 9.77 and 15.14 mg/mL, respectively. In both enzymes including GPT and GOT, the isolated compounds (*p < 0.01*) significantly inhibited greater than the compared standard gallic acid and tannic acid. Interestingly, among both isolated compounds, compound 1 showed (*p < 0.01*) significant superiority over compound 1. The effects of isolated oligomeric proanthocyanidins 1 and 2 showed (*p < 0.01*) inhibition of cellulase with EC_50_ of 15.85 and 23.98 mg/mL, respectively (Figure 4), even though compound 1 has strong affinity over compound 2 to bind cellulase enzyme. Furthermore, these compounds exhibited EC_50_ at a lower value when compared with gallic acid (89.13 mg/mL) and tannic acid (109.65 mg/mL). Consequently, these might have an effect on fiber digestibility.

Phenolic compounds exhibited the inhibition effect due to their anti-microbial properties and additional metabolites generated during the fermentation process (Kamra et al., 2022). Similar trends were observed in rumen kinetics by using phenolic/methanolic leaf extracts of *Ficus* species (Koli et al., 2022) and *Anogeissus pendula* (Singh et al., 2022; Lata et al., 2023), as well as other phenolic plant extracts (Patra et al., 2006; Dhanasekaran et al., 2020; Kamra et al., 2022). It is believed that plant phenolic compounds could be a strong antioxidant after breaking down from plant extract, resulting in the reduction of ruminal enzymatic activities (Ishida et al., 2015). The inhibitory effects of forage legume phenolic extracts on cellulose digestion (McAllister et al., 2005) and phenolic acids’ decreasing activities of rumen enzymes *in vitro* (Berchez et al., 2019) help to support our findings on the reduction of ruminal cellulase activity. The observed decrease in ruminal GOT, GPT, and cellulase activity in ruminal enzymes signifies potential benefits for animal nutrient utilization (Lata et al., 2023). This reduction implies improved amino acid metabolism and enhanced protein synthesis. Simultaneously, the lower cellulase activity suggests a more controlled breakdown of cellulose, pointing toward improved fiber utilization in the rumen and overall enhanced nutrient absorption during digestion.

**FIGURE 5:**
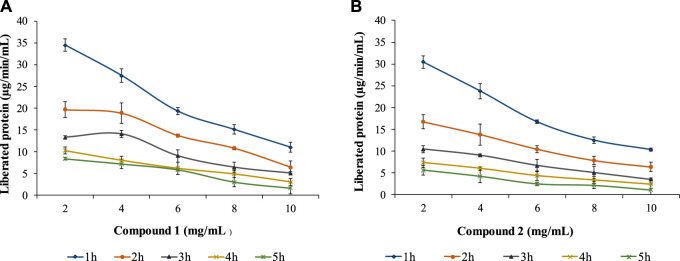
Effects of compound 1 **(A)** and compound 2 **(B)** on ruminal protease with different concentrations and incubation times.

The study also examined compounds 1 and 2 against the ruminal protease with different concentrations (2, 4, 6, 8, and 10 mg/mL) and incubation times (1, 2, 3, 4, and 5 h) for both compounds in the substrate (Figure 5). The proteolysis activity decreased with the time and concentration of polyphenols (proanthocyanidins). Both compounds showed similar trends even though compound 1 was found to be less effective than compound 2. Compound 1 could lower the activity to 11.024 μg/min/mL at a maximum concentration (10 mg/mL) at 1h and 1.654 μg/min/mL in 5 h in comparison to compound 2 which allowed the protein liberation in 10.34 μg/min/mL and 1.13 μg/min/mL in similar conditions.

A similar observation of lowering the concentration of liberated rumen protein in the presence of isolated compounds 1 and 2 with elevated concentrations and incubation times with phenolic extracts of *Lotus pendunculatus* (McNabb et al., 1996), tannin-rich forage leaves (Aerts et al., 1999; Bhatta et al., 2012), proanthocyanidins obtained from *Ficus* (Koli et al., 2022) and *Anogeissus* species (Singh et al., 2022; Lata et al., 2023), phenolics extracted from pomegranate skin (Abarghuei et al., 2021). The movement of protein from the stomach to the intestine, where the animal can use it, depends on how strongly it binds. This binding is affected by the different structures of certain compounds and the nature of the proteins, causing variations in the overall process (Waghorn et al., 1987). The interaction of proteins and phenolic compounds is a complex phenomenon, and it is hypothesized that the presence of proanthocyanidins/phenolics enhances this flow. In general, various phenolic compounds impact protein-phenolic interactions based on factors like molecular weight, methylation, hydroxylation, glycosylation, and hydrogenation. The binding affinity between polyphenols and proteins rises with the molecular size of polyphenols (Ozdal et al., 2013). The decrease in protease activities could be due to the steric hindrance at interaction sites of protease and receptors. The basic route of proteolysis inhibition by phenolic compounds is based on interference with the interaction of enzyme substrates (McManus et al., 1981). Therefore, proanthocyanidins may yield positive effects when binding with proteins (Aerts et al., 1999; Patra and Saxena, 2011) or negative effects when reducing ruminal digestion without binding to proteins, especially concerning hemicellulose (Tanner et al., 1994; Jonker and Yu, 2017). The compounds isolated from F. glomerata leaves hold promise as a natural and sustainable additive for animal feed, with the potential to enhance nutrition, minimize health risks, and reduce environmental pollution. Novel animal feed formulations incorporating these compounds can be developed, and their effects on animal growth, health, and overall wellbeing should be thoroughly evaluated through controlled feeding trials. Furthermore, scientific investigations and conclusive studies are warranted to explore the potential of these compounds in mitigating environmental pollution by minimizing the excretion of harmful substances in animal waste.

## 4 Conclusion

This research not only focused on the characterization of two novel compounds derived from leaves of *F. glomerata* leaves but also strategically employed a dual evaluation approach using the *in silico* study. The structure-activity relationships are very significant in depicting the nutritional impacts of proanthocyanidins or any polyphenols. The observed significant inhibitory effects on rumen enzyme activity underscore the potential of these compounds to enhance feed conversion ratios and advance drug development. Furthermore, the findings significantly contribute to our molecular understanding of proanthocyanidins, emphasizing their candidacy as potential drugs and enriching our knowledge of their role in rumen physiology. The current investigations represent just a small initial step in this direction.

## Data Availability

The datasets presented in this study can be found in online repositories. The names of the repository/repositories and accession number(s) can be found in the article/Supplementary Material.
