# Supplementary material for: Proanthocyanidins isolated from the leaves of Ficus glomerata evaluated on the activities of rumen enzymes: in vitro and in silico studies
Source: Front Chem. 2024 Feb 6;12:1359049. doi: 10.3389/fchem.2024.1359049 (PMC10877006; doi:10.3389/fchem.2024.1359049)
Supplement: Supplementary file 1 [file DataSheet3.docx]

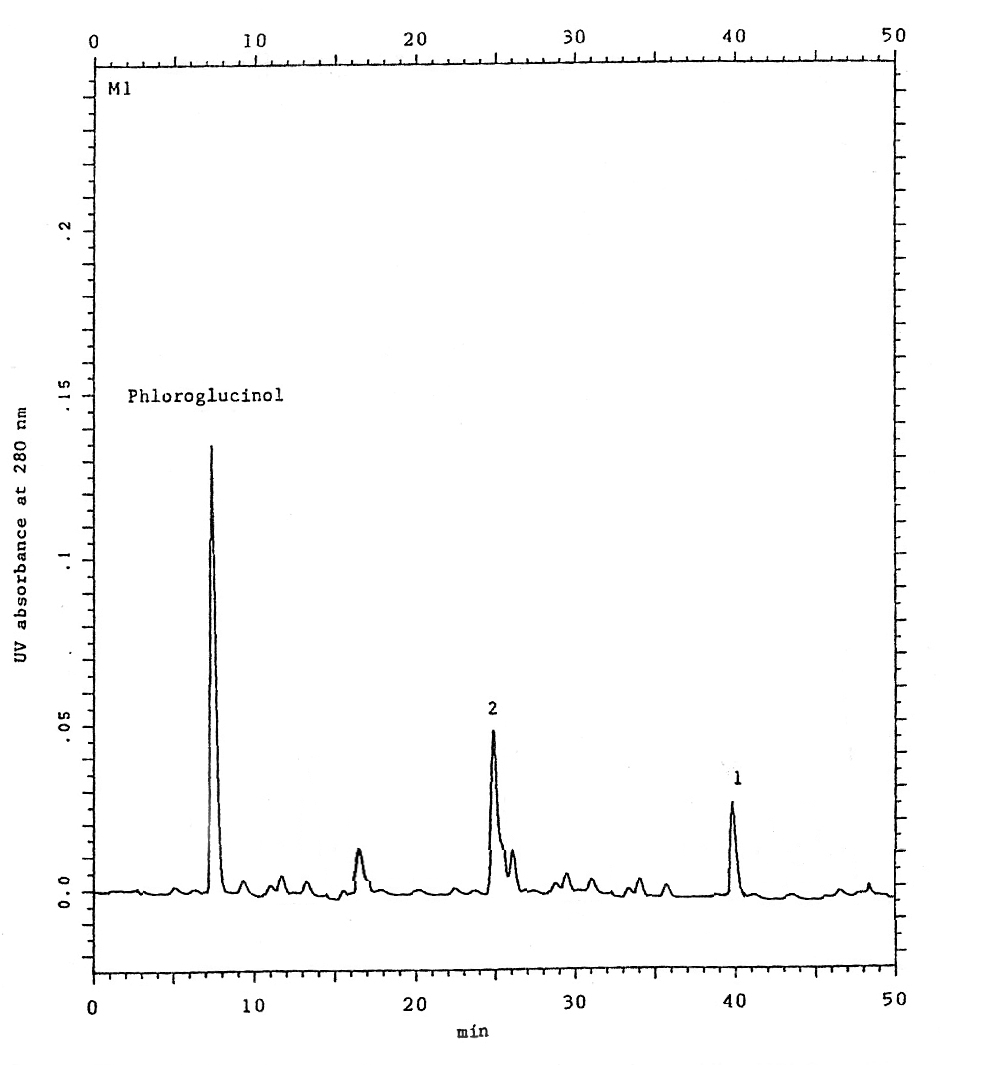


**HPLC chromatograms for Compound 1 and 2**


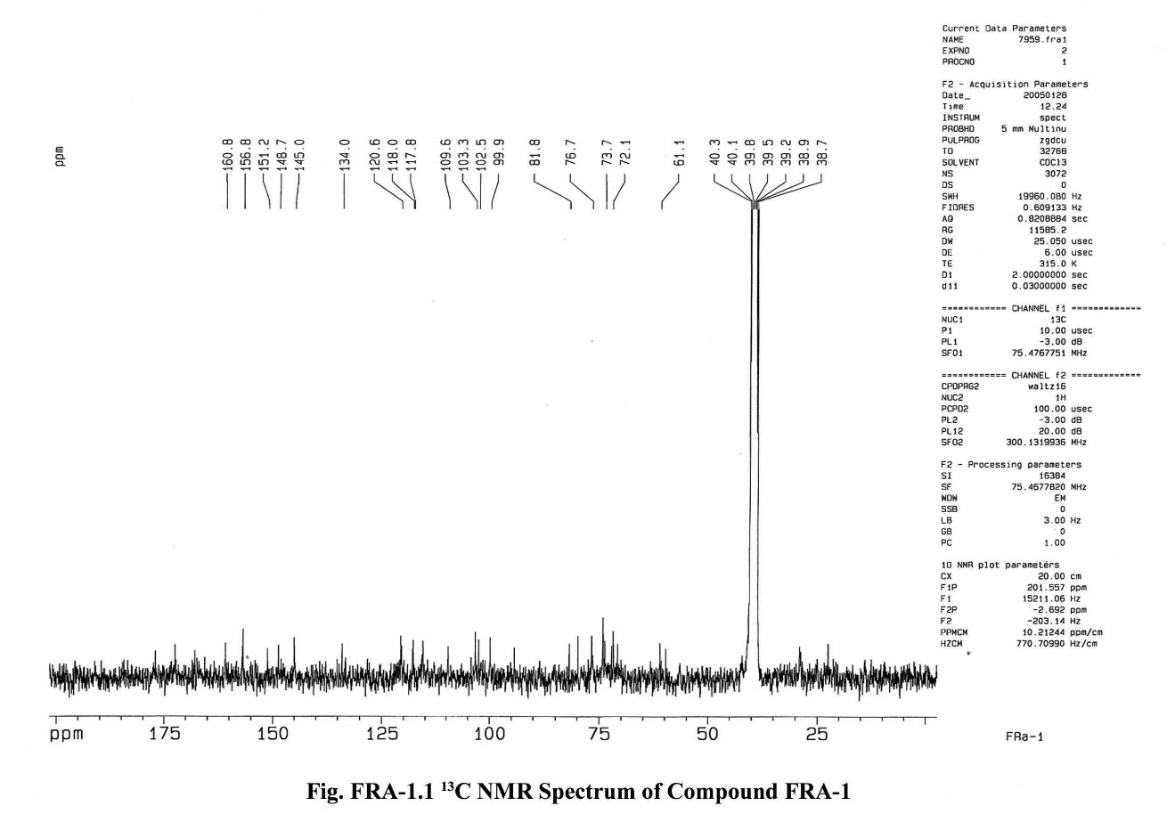


**^13^C NMR Spectra of Compound 1**


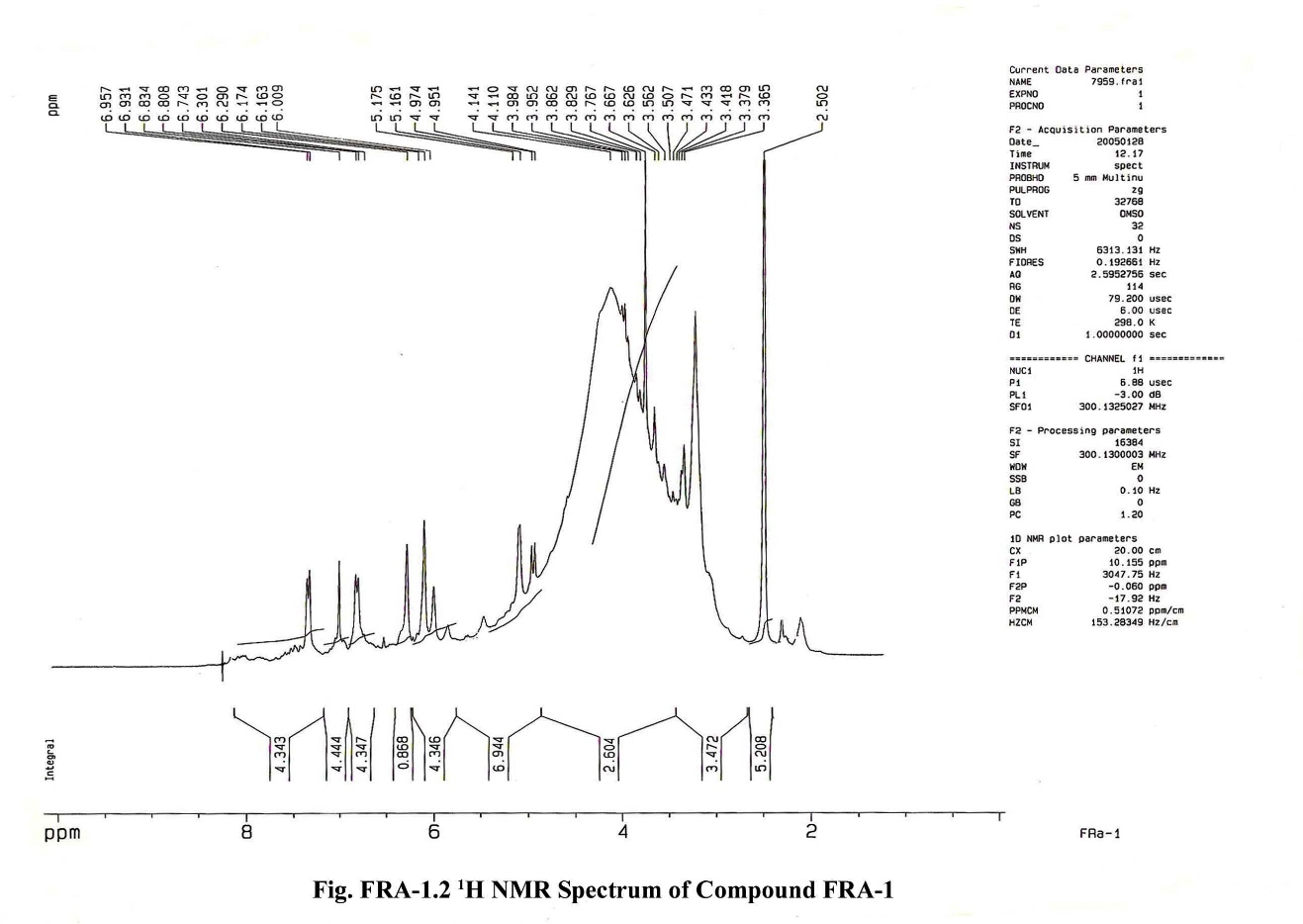


**^1^H NMR Spectra of Compound 1**


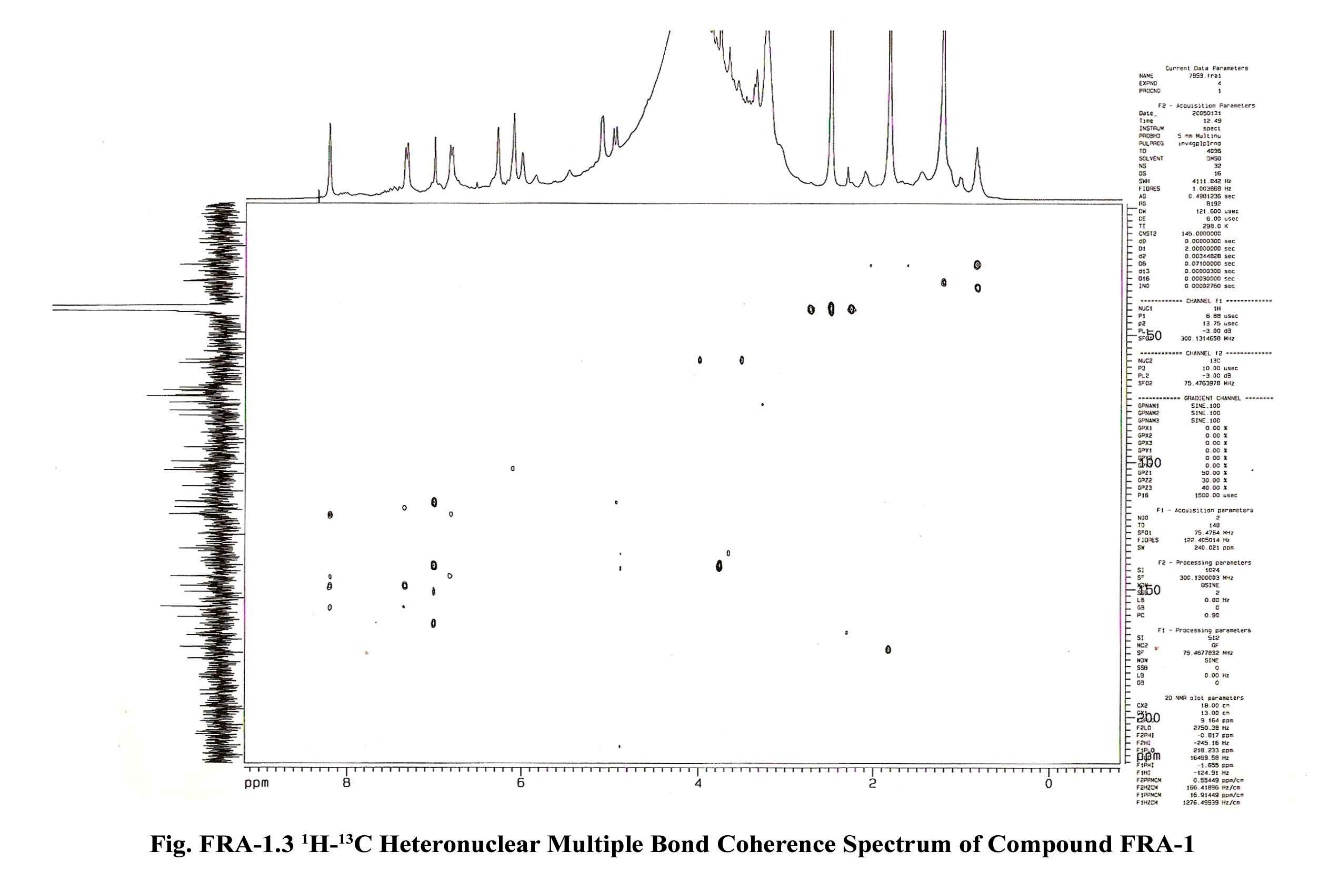


**^1^H-^13^C Heteronuclear Multiple Bond Coherence (HMBC) Spectrum of Compound 1**


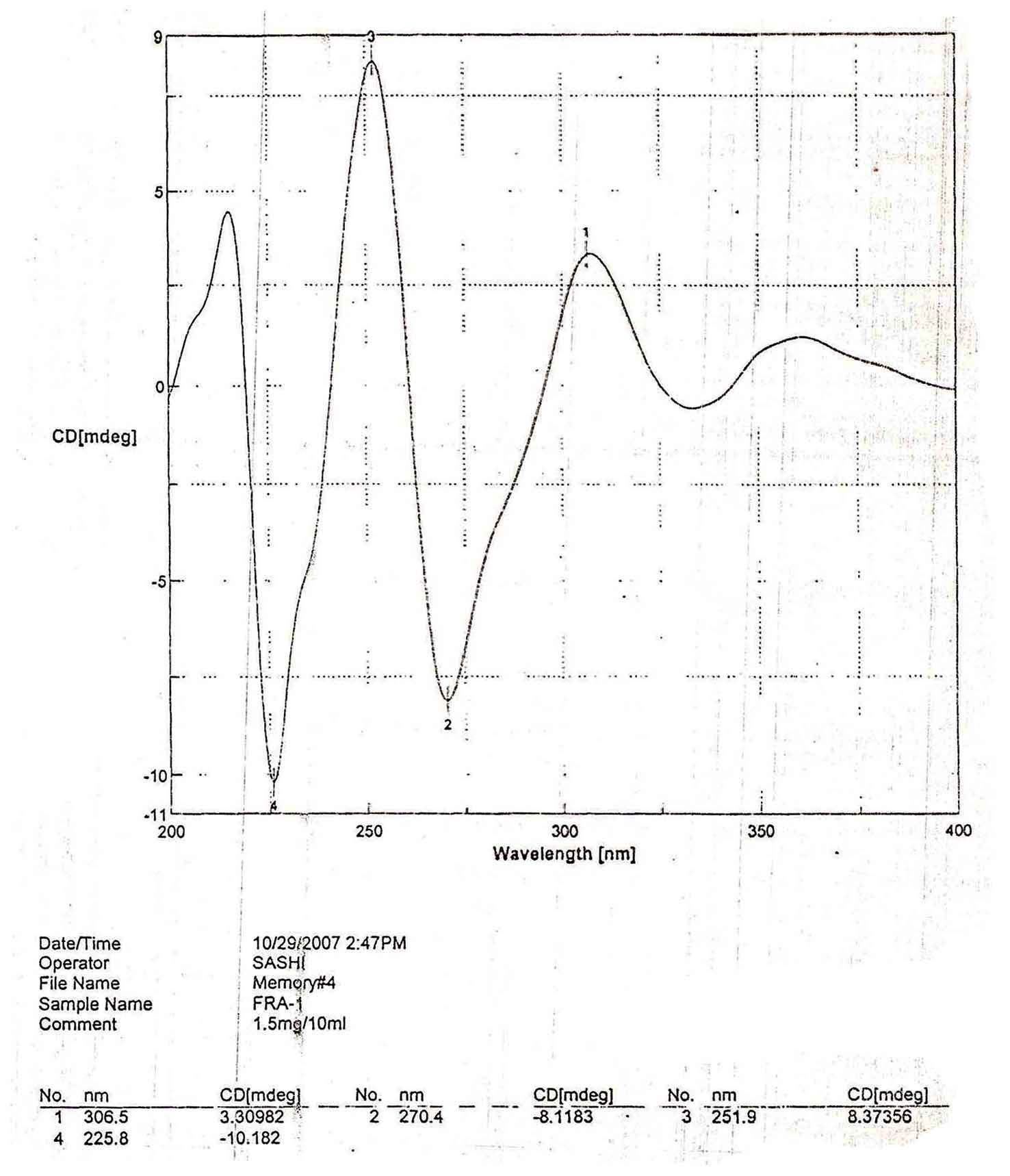


**CD Spectrum of Compound 1**

**
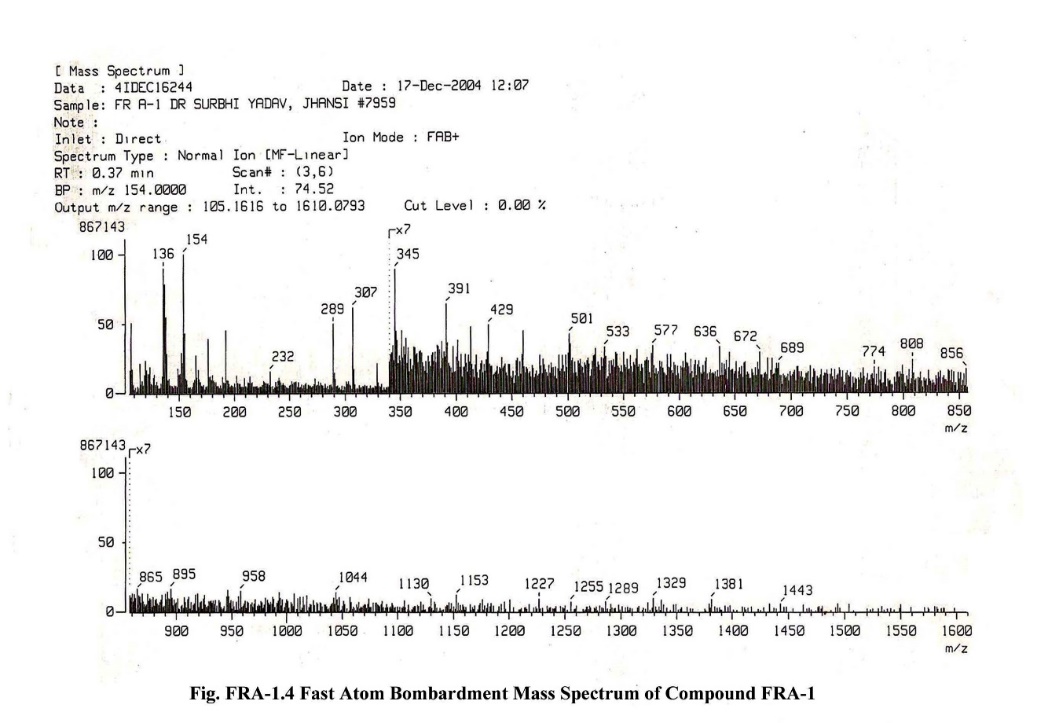
**

**Fast Atom Bombardment Mass Spectrum of Compound 1**


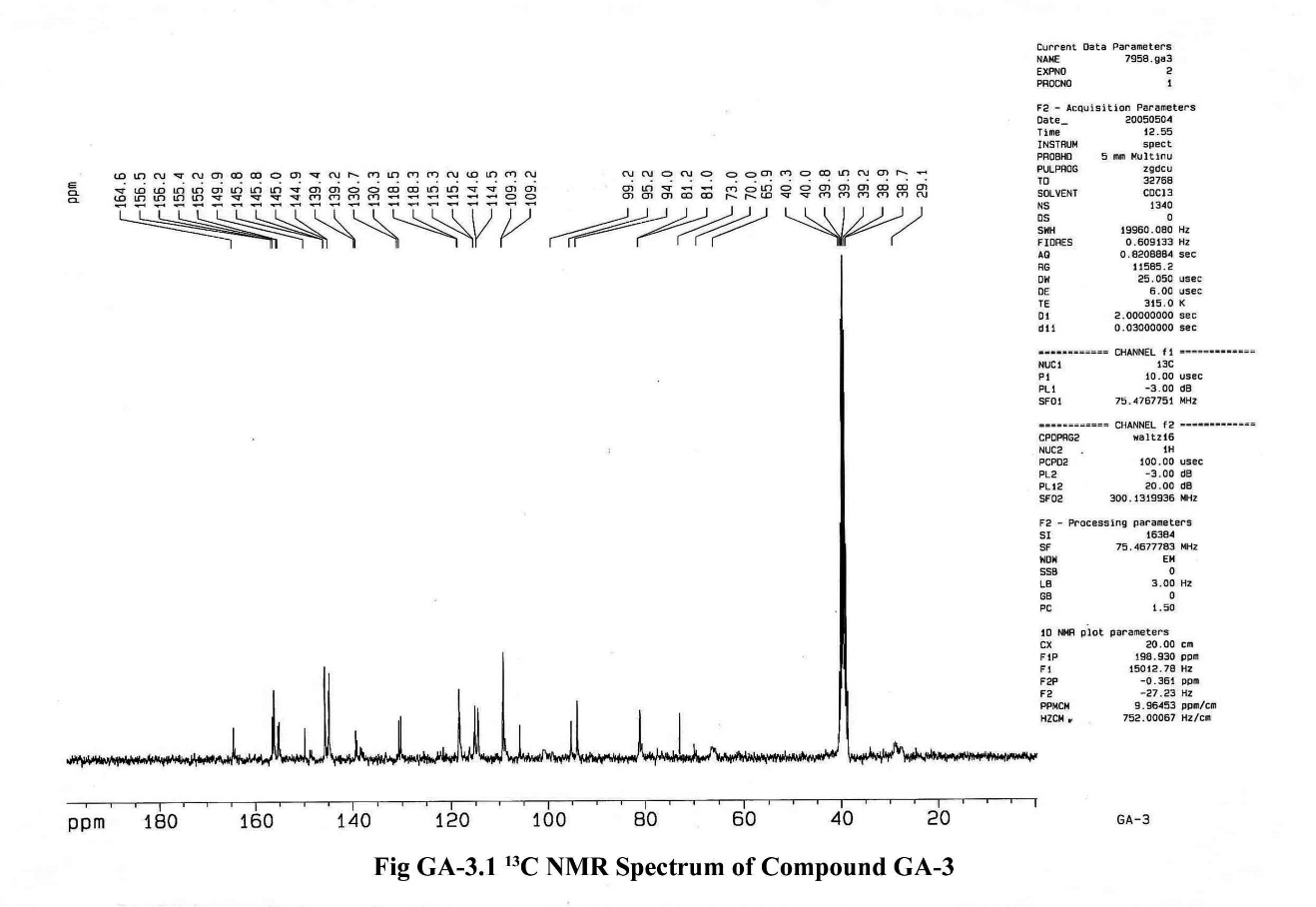


**^13^C NMR Spectra of Compound 2**


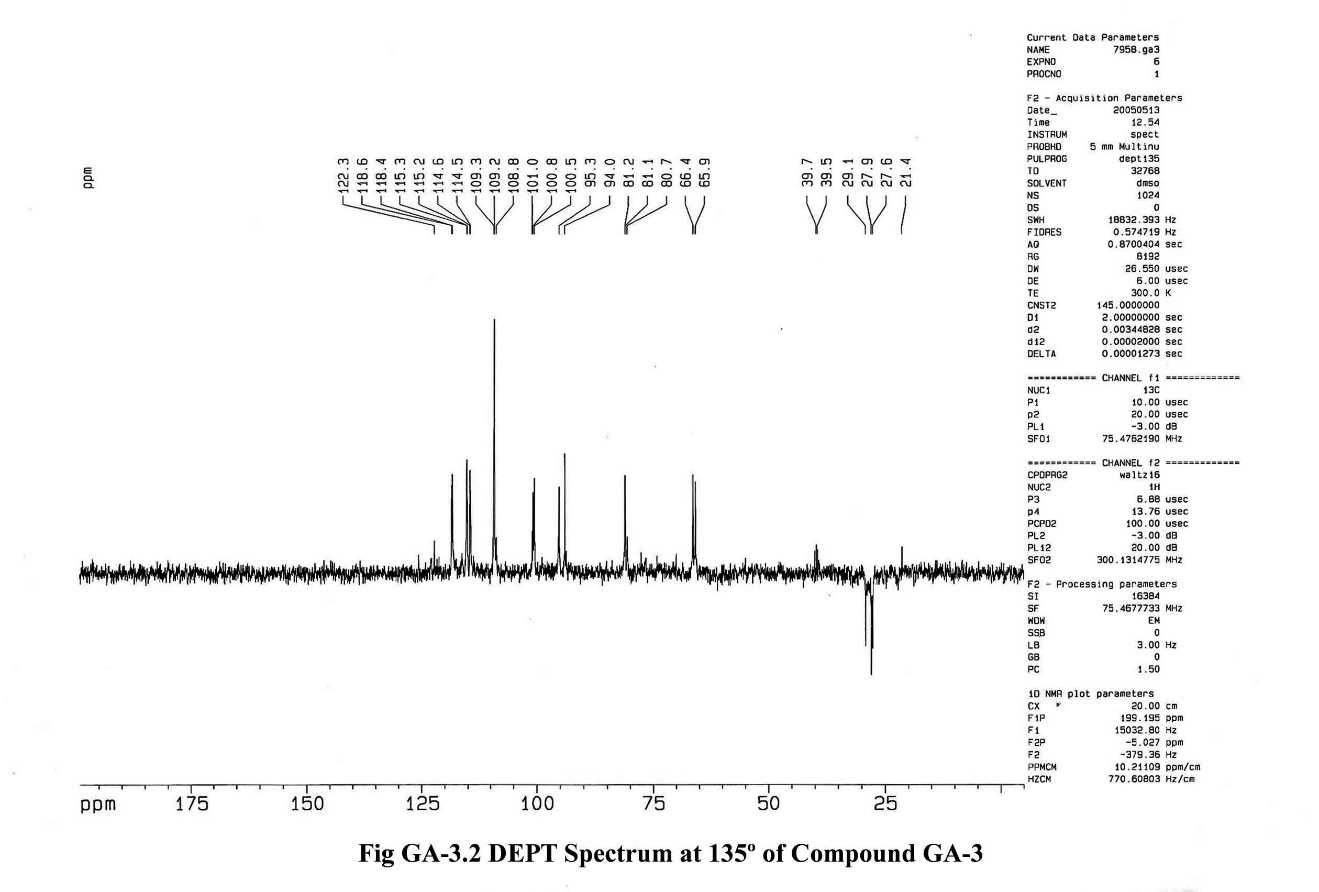


**DEPT Spectrum at 135^o^ of Compound 2**


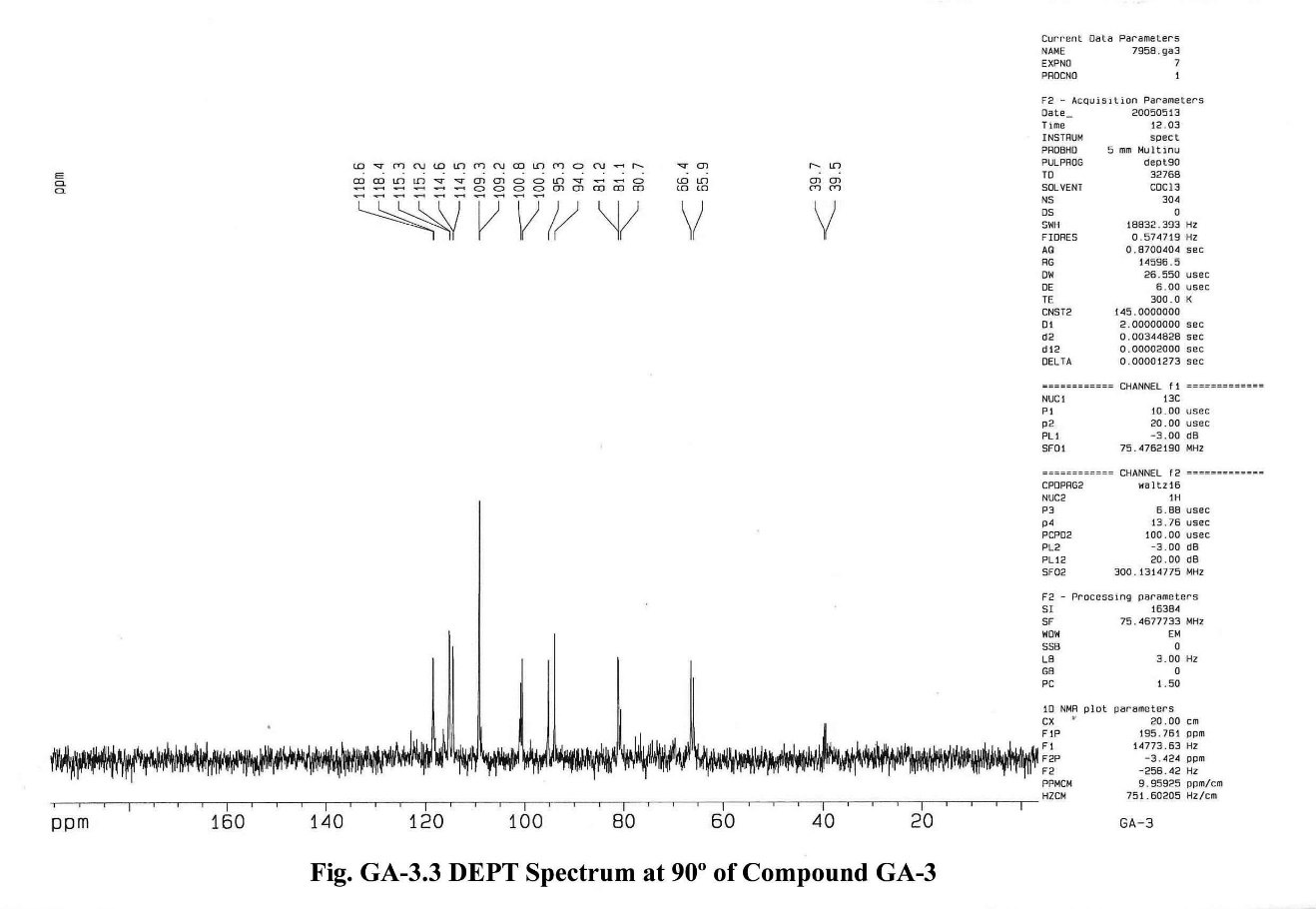


**DEPT Spectrum at 90^o^ of Compound 2**


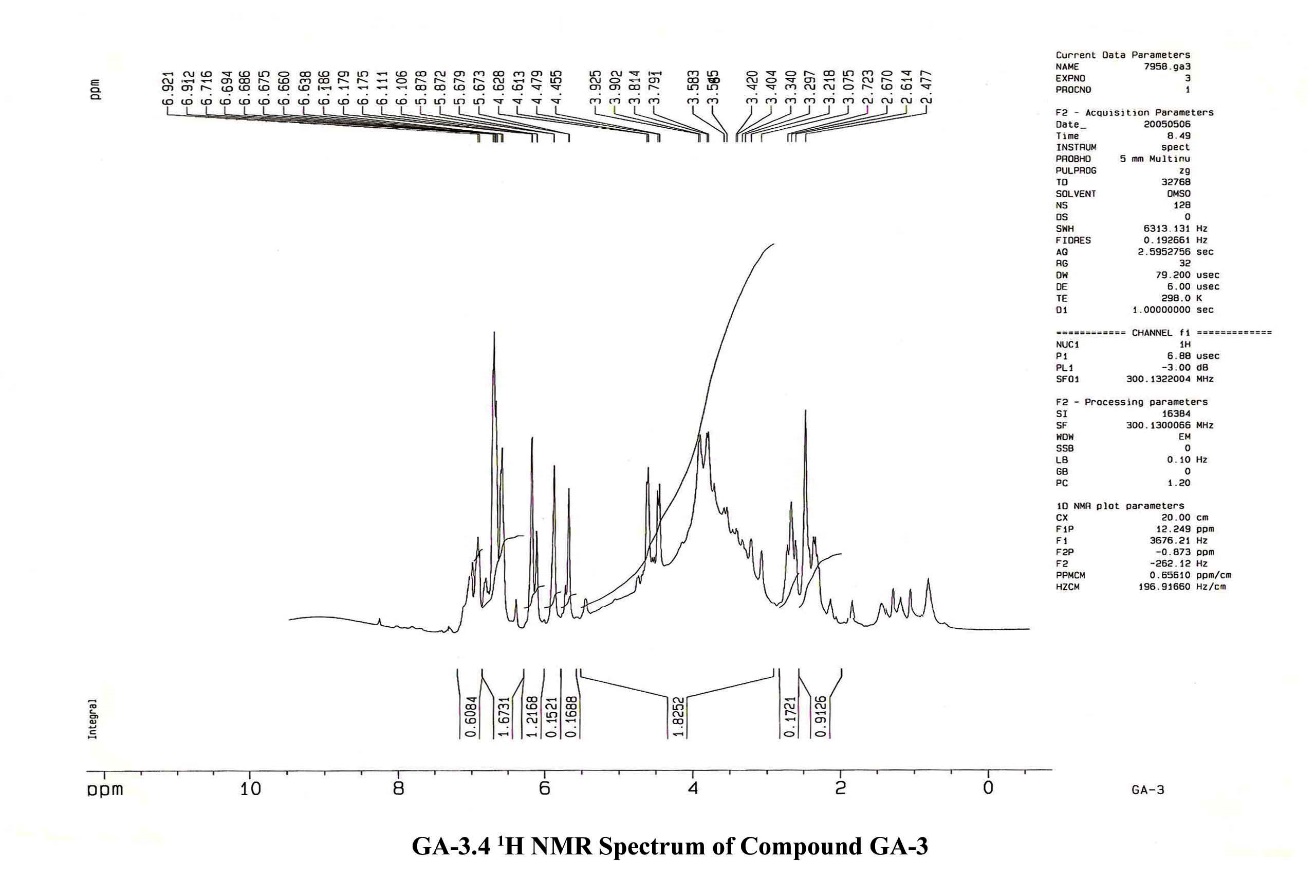


**^1^H NMR Spectra of Compound 2**


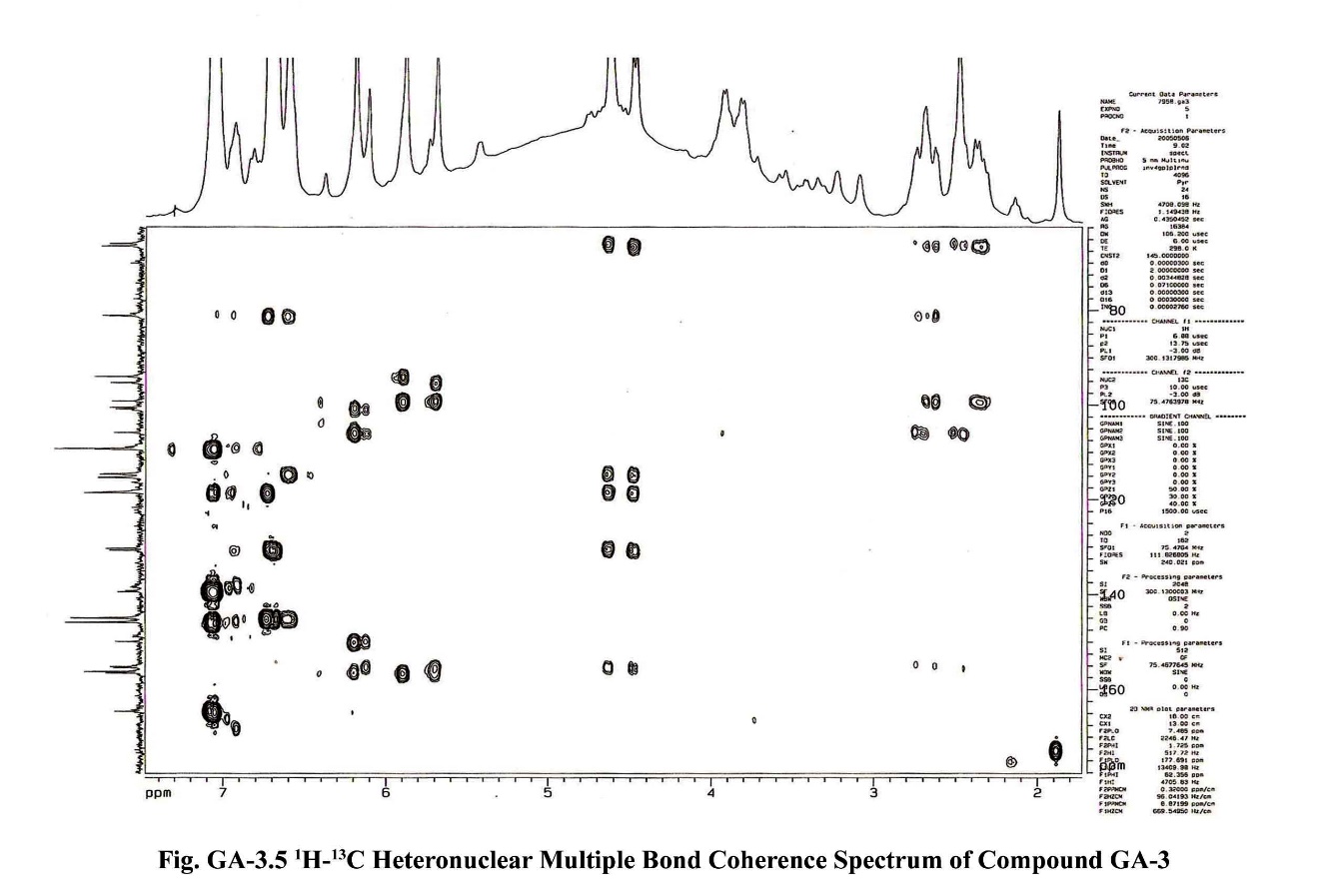


**^1^H-^13^C Heteronuclear Multiple Bond Coherence (HMBC) Spectrum of Compound 2**


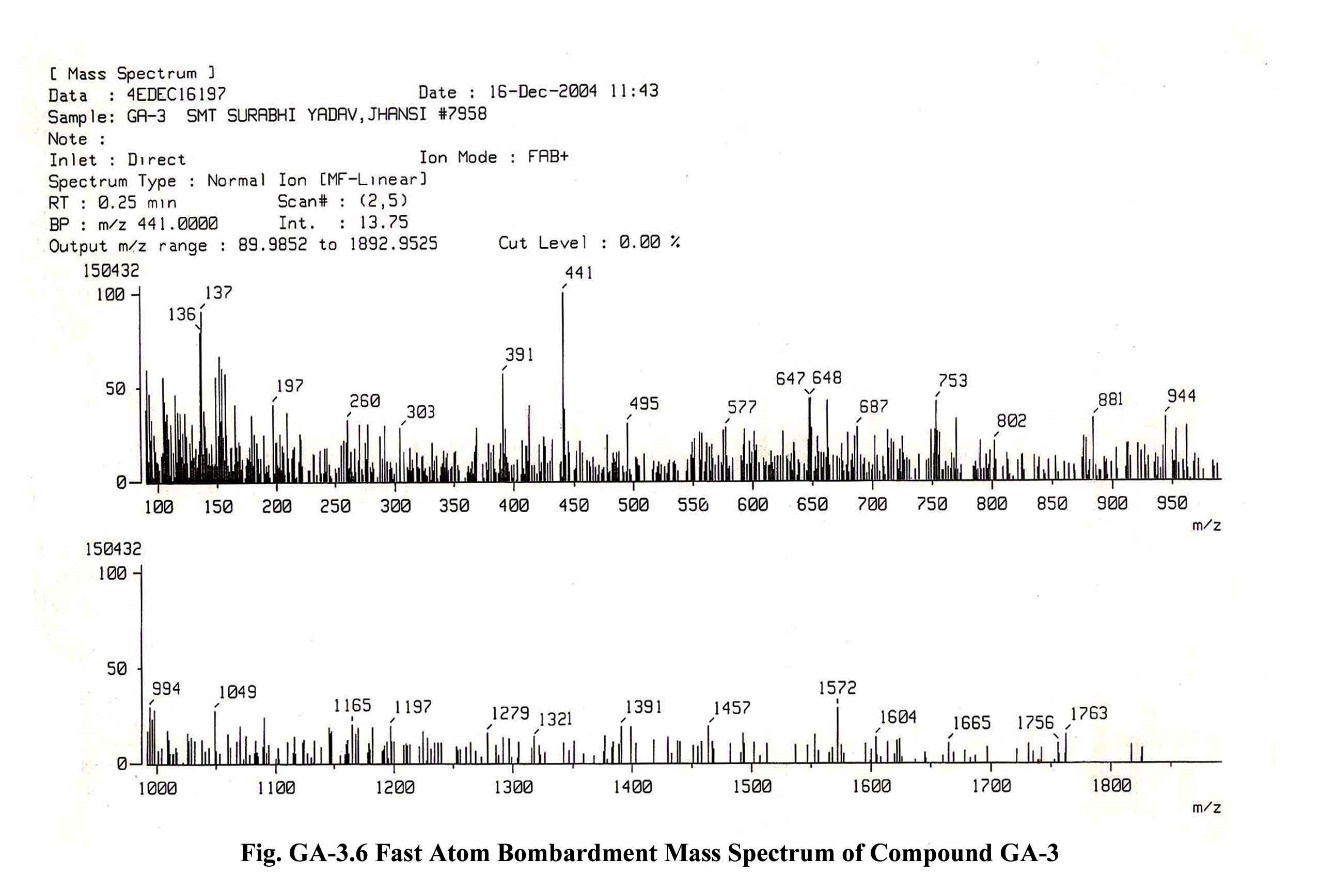


**Fast Atom Bombardment Mass Spectrum of Compound 2**
