# Supplementary figures and images for: Proanthocyanidins isolated from the leaves of Ficus glomerata evaluated on the activities of rumen enzymes: in vitro and in silico studies
Source: Front Chem. 2024 Feb 6;12:1359049. doi: 10.3389/fchem.2024.1359049 (PMC10877006; doi:10.3389/fchem.2024.1359049)

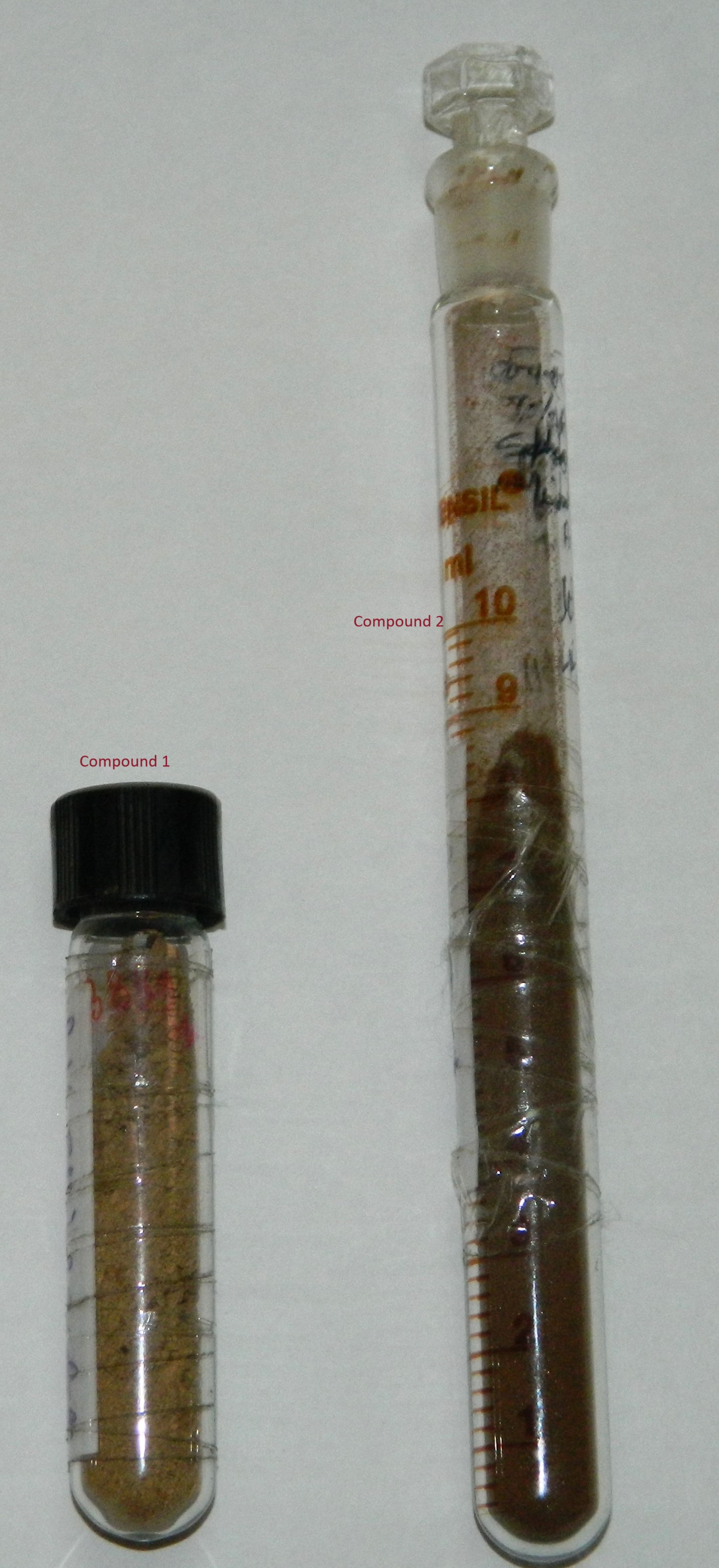

Supplement: Supplementary file 2 [file Image2.JPEG]

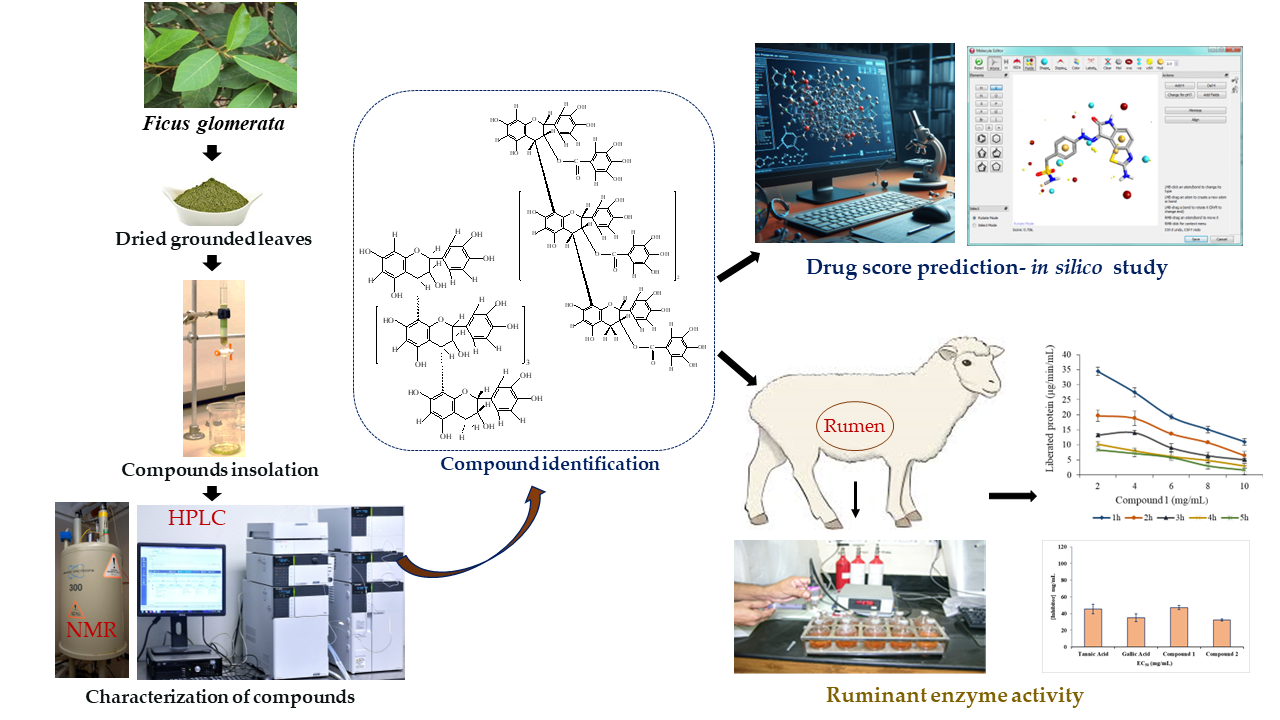

Supplement: Supplementary file 3 [file Image1.PNG]
